# Supplementary material for: Activity and Stability of Panx1 Channels in Astrocytes and Neuroblastoma Cells Are Enhanced by Cholesterol Depletion
Source: Cells. 2022 Oct 14;11(20):3219. doi: 10.3390/cells11203219 (PMC9600160; doi:10.3390/cells11203219)
Supplement: Supplementary file 1 [file cells-11-03219-s001.zip › cells-1932037-supplementary.pdf]

SUPPLEMENTAL INFORMATION:

Activity and Stability of Panx1 Channels in Astrocytes and Neuroblastoma Cells Are Enhanced by Cholesterol Depletion

Antonio Cibelli [1,*,†], Eliana Scemes [2] and David C. Spray [1]

[1] Department of Neuroscience, Albert Einstein College of Medicine, Bronx, NY 10461, USA
[2] Department of Cell Biology and Anatomy, NY Medical College, Valhalla, NY10595, USA
* Correspondence: antonio.cibelli@uniba.it;
† Present Address: Department of Biosciences, Biotechnologies and Biopharmaceutics, University of Bari Aldo Moro, Via Orabona 4, 70125 Bari, Italy; Tel. +39-0805443335

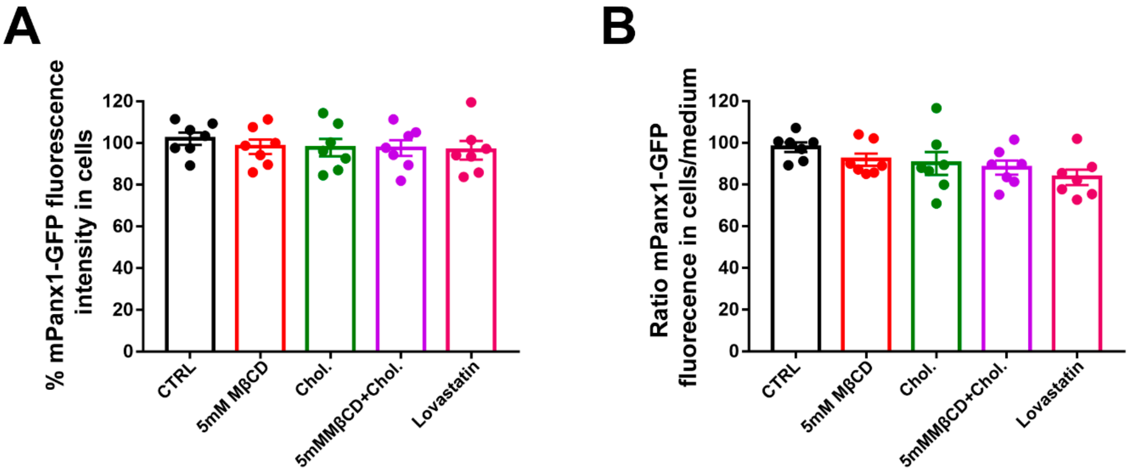

Supplemental Figure S1. Effects of treatment with MβCD and lovastatin on mPanx-1-GFP expression. mPanx-1-GFP transfected N2a cells were incubated with cyclodextrin (5 mM MβCD for 60 min at 37°C), lovastatin (5 µM, 12 hr) in serum-free DMEM, cholesterol (2h, 37°C) or left untreated (CTRL). (A) Histograms showing mPanx-1-GFP fluorescence intensity in cells expressed as a percentage relative to that of untreated cells (CTRL), considered as 100 %. Note that treatments did not effect mPanx1-GFP expression intensity in cells. Each point on the graphs represents relative mPanx1-GFP fluorescence changes recorded from all cells present in a field of view obtained from at 3 independent experiments. (B) Ratios of fluorescence intensity of mPanx-1-GFP in cells to medium. Note that the ratios of intensity are similar. ANOVA test followed by Dunnett’s test.

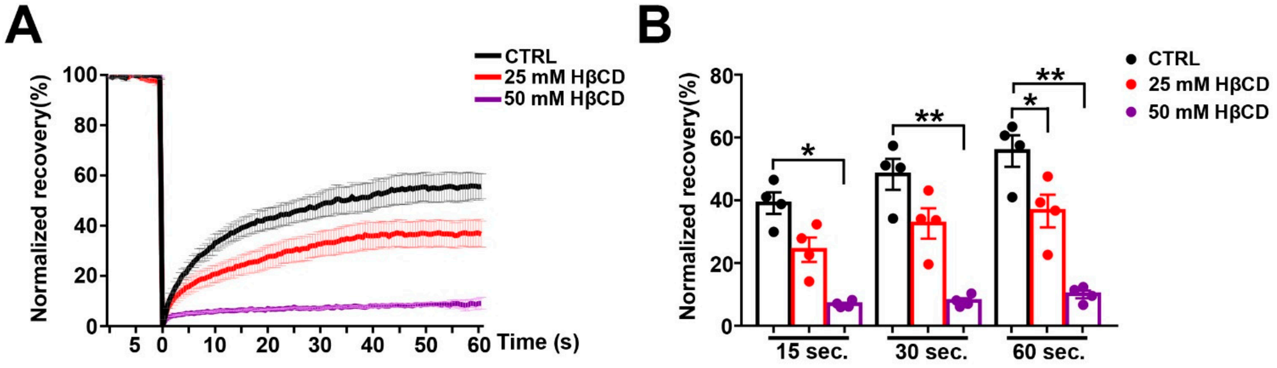

Supplemental Figure S2. Effects of 2-Hydroxypropyl-β-cyclodextrin on the lateral diffusion of mPanx-1-GFP. N2a cells transiently expressing mPanx-1-GFP were subjected to treatments with 25 mM and 50 mM of 2-Hydroxypropyl-β-cyclodextrin (HβCD) or left untreated (CTRL). FRAP experiments were conducted on live cells at 30°C. (A) FRAP recovery curves for control cells and with 25 mM and 50 mM of HβCD. (B) Histograms of normalized FRAP recovery at 15, 30 and 60 sec after photobleach. n=7–10 per plasma membrane domain, data collected over three independent repeats. P values obtained from ANOVA test followed by Dunnett's test. (*: p<.05; **p<.01).
